# 

**Published:** 1896-05

**Authors:** 


					﻿Vol. XVI.
MAY, 1896.
No. 5^
THE
HOMOEOPATHIC pHY£lClA]\I,
A Monthly Journal of Homoeopathic Materia Medica
and Clinical Medicine.
“He it a freeman whom truth makes free, and all are slaves beside."
“Seek the Truth: come whence it may, cost what it will."
♦ - • '
EDITED AND PUBLISHED BY
WALTER M. JAMES, M. D.
PHILADELPHIA:
JSTo. 1231 LOCUST STREET.
LONDON
ALFRED HEATH & CO., Sole Agents fob Great Britain,
114 Ebury Street, Eaton Square.
'Yearly Subscription, $2.50, invariably in advance. Single numbers, 25 etc.
Entered at the Philadelphia Po.t-Offlee aa Second-Clan Mail Matter.
				

